# Stimulation-mediated reverse engineering of silent neural networks

**DOI:** 10.1152/jn.00100.2023

**Published:** 2023-05-24

**Authors:** Xiaoxuan Ren, Ilhan Bok, Adam Vareberg, Aviad Hai

**Affiliations:** ^1^Department of Biomedical Engineering, University of Wisconsin-Madison, Madison, Wisconsin, United States; ^2^Department of Electrical and Computer Engineering, University of Wisconsin-Madison, Madison, Wisconsin, United States; ^3^Wisconsin Institute for Translational Neuroengineering (WITNe), Madison, Wisconsin, United States

**Keywords:** connectivity, microelectrode arrays, reverse engineering, supervised learning

## Abstract

Reconstructing connectivity of neuronal networks from single-cell activity is essential to understanding brain function, but the challenge of deciphering connections from populations of silent neurons has been largely unmet. We demonstrate a protocol for deriving connectivity of simulated silent neuronal networks using stimulation combined with a supervised learning algorithm, which enables inferring connection weights with high fidelity and predicting spike trains at the single-spike and single-cell levels with high accuracy. We apply our method on rat cortical recordings fed through a circuit of heterogeneously connected leaky integrate-and-fire neurons firing at typical lognormal distributions and demonstrate improved performance during stimulation for multiple subpopulations. These testable predictions about the number and protocol of the required stimulations are expected to enhance future efforts for deriving neuronal connectivity and drive new experiments to better understand brain function.

**NEW & NOTEWORTHY** We introduce a new concept for reverse engineering silent neuronal networks using a supervised learning algorithm combined with stimulation. We quantify the performance of the algorithm and the precision of deriving synaptic weights in inhibitory and excitatory subpopulations. We then show that stimulation enables deciphering connectivity of heterogeneous circuits fed with real electrode array recordings, which could extend in the future to deciphering connectivity in broad biological and artificial neural networks.

## Graphical abstract

**Figure:**
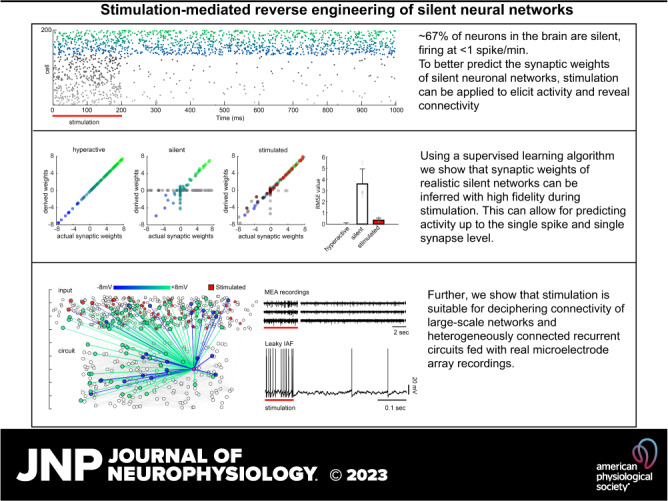

## INTRODUCTION

Deciphering the function of neurobiological networks hinges on proper determination of connectivity at the single neuronal level (1–4). Uncovering the specific circuit connectivity and how it mediates neuronal firing can then lead to more effective therapies for brain disorders (5–10). Different methodologies have been used to derive neural coupling at the neuronal level (11–16) and at the brain regional level (17–19). Current efforts involve reconstructing highly detailed structural connections (11, 20, 21) or deriving putative monosynaptic connections from large-scale electrophysiological recordings (15, 22) and optical imaging (2, 16, 23, 24). Notwithstanding the growing availability and increased precision of neurological imaging modalities, multiplexed electrophysiological recordings of action potentials by microelectrode arrays (MEAs) are still the mainstay input for decoding connectivity at microcircuit resolution (14, 25, 26). MEAs provide readouts at increasingly larger scales, with recent probes able to provide in vivo recordings of up to thousands of units in parallel (27–30).

Most studies rely primarily on active units to derive connectivity and largely exclude nonfiring or minimally active neurons. However, numerous groups present evidence that more than half of the neurons in the brain are silent, firing at very low frequencies (31, 32) and having highly specialized receptive fields (33–35). Minimally active neurons do not fire spontaneously or participate in oscillatory activity but instead usually fire at rates of 1 spike/min or less (36–38), with some groups reporting frequencies of as low as 0.001 Hz (39) depending on the brain region and task studied. The proportion of silent neurons in a typical brain tissue varies, ranging between at least 10–20% in the sensory cortex (38, 39) and as much as 66% in the motor cortex (36). Low firing rates limit the accuracy of network reconstruction, because of the inherently low information content at baseline conditions. Electrical stimulation (40, 41), sensory stimulation (42, 43), and neuropharmacological manipulations (44–46) are often used in neurobiological research to perturb a system above baseline and could therefore activate silent neurons and thus improve the inference of network connectivity.

In this work we demonstrate a supervised learning method to analyze the use of stimulation for determining connectivity between postsynaptic neurons and heterogeneous populations of both silent and active presynaptic neurons. We test our method on simulated spike trains and apply it on experimental multielectrode array data of rat cortical neurons, using circuits of leaky integrate-and-fire (LIF) neuronal models. We show that repetitive stimulation epochs can evoke activity that is sufficient for determining the synaptic weights of the entire neuronal population. We characterize the performance of our algorithm for determining weights from excitatory, inhibitory, and unconnected neurons in the population and compare the ability of the method to predict spike trains. This approach presents a new platform to increase the performance of learning algorithms for reconstructing neuronal networks and for using neuronal stimulation to decipher large-scale brain circuitry.

## METHODS

### Simulated Neuronal Population Spike Trains

We generated spike trains for populations of neurons (size ranging between 200 and 2,000 cells) recorded during stimulus response. Sixty-six percent of the spike trains corresponded to “silent” neurons [firing at 1 spike/min, or 0.017 Hz (35, 47)], and the rest corresponded to responsive neurons [mean firing rate was 20 Hz (48, 49)]. For feedforward perceptron networks spikes were generated by sampling randomly using a uniform distribution with a probability of 1/f, and for recurrent neuronal circuits spike distribution was lognormal (µ = 3.7 Hz, σ = 3.5 Hz). In addition to the above condition, we compared results using simulated spike trains from a population with no silent neurons, where all the 200 units fired at 20 Hz (hyperactive network).

### Feedforward Integrate-and-Fire Model and Connectivity

For feedforward perceptron networks we simulated postsynaptic neurons as simple integrate-and-fire (IAF) neurons receiving inputs from 100 out of the total population spike trains. Eighty inputs were set to be excitatory (having positive weights), and 20 inputs were set to be inhibitory (having negative weights) (50). Input weights ranged between [−8, 8] mV, sampled randomly with a uniform distribution. Spikes in the simple IAF model were generated by summation of the inputs at each time step (1-ms intervals) using

(*1*) $$V=\sum_{i=1}^{n}x_{i}w_{i}y\;=\;1\text{, }V>\text{threshold}y\;=\;0\text{, }V\leq \text{ threshold}$$

where *V* is the membrane potential of the postsynaptic neuron, *x_i_* is the input from population neuron *i*, *w_i_* is the weight of input *i*, *n* = total number of neurons, threshold = 20 mV (with resting membrane potential = 0 mV), and *y* is the spike output of the postsynaptic neuron. All models were constructed in MATLAB 2020a (MathWorks Inc, Natick, MA).

### Leaky Integrate-and-Fire Recurrent Circuits

For recurrent circuits we used leaky integrate-and-fire (LIF) neuronal models to enable tonic excitability in response to input (51). Postsynaptic spike at time *t* + Δ*t* was determined by a threshold and reset mechanism:

(*2*) $$V_{\text{m}}\left(t+\Delta t\right)=\;V_{\text{m}}\left(t\right)+\;\Delta t\cdot \frac{-\left[V_{\text{m}}\left(t\right)\;-\;V_{\text{e}}\;\right]+\;I_{\text{m}}\cdot \;R_{\text{m}}}{\tau_{\text{m}}}$$

where *V*_m_ is the membrane potential, *V*_e_ is the resting potential (−75 mV), *C*_m_ is the membrane capacitance (100 pF), and *R*_m_ is the input resistance (10 MΩ), with a time constant τ_m_ = *C*_m_ × *R*_m_ = 1 ms. *I*_m_ is the membrane current defined as sum total of presynaptic current inputs:

(*3*) $$I_{\text{m}}=I_{\text{post}}\overset{}{\underset{i}{\sum }}w_{i}\;\times \;{\text{spike}}_{i}\;$$

contributed by each presynaptic cell *i* with weight *w_i_*, active if spike*_i_* = 1 and with postsynaptic current *I*_post_ = 1 nA. A postsynaptic spike is generated if *V*_m_ > *V*_t_ (where *V*_t_ is threshold voltage), and *V*_m_ is reset to *V*_reset_. 

### Circuit Connectivity

Circuits of interconnected LIF neurons were constructed with nonrandom connectivity distribution: neurons were connected with probabilities *P* = 0.13 for unidirectional connections and *P*_rep_ = 0.06 for reciprocal connections (52). Reciprocal connections were 1.5 times stronger than unidirectional connections. Each neuron in the circuit had a total presynaptic neural population of 120 cells with excitatory-to-inhibitory weight ratio of 4:1 and weights a range of [−8, 8] mV. Firing rate distribution for circuit cells was lognormal (µ = 0.8703 Hz, σ = 0.8749 Hz).

### Deriving Connectivity

We used the spike trains of the postsynaptic neuron and the presynaptic population as ground-truth data for deriving connection weights with a perceptron algorithm (53, 54). At each iteration of the algorithm, the derived connection weights were updated using the perceptron learning rule:

(*4*) $$\Delta w_{i}=\text{lr }\left(y-y\prime \right)x_{i}$$

**Figure 1.**
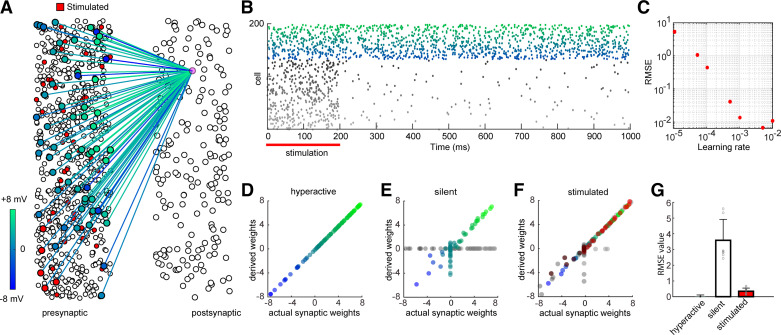
Stimulation-mediated derivation of synaptic weights of feedforward neuronal population with silent cells. *A*: schematic diagram of a neuronal population of presynaptic neurons forming synapses with an integrate-and-fire postsynaptic neuron (purple), with weights ranging between −8 and 8 mV; 66% of the neurons in the population were silent (firing rate < 0.01 Hz), and the rest were active (firing rate ∼20 Hz). In the stimulated condition, 25% of the neurons were stimulated (firing rate ∼60 Hz). The color coding is maintained for the rest of the figure. The postsynaptic neuron received excitatory and inhibitory inputs with a 4-to-1 ratio. *B*: spike raster of the population spike trains over 1,000 ms. The top 34% of the trains correspond to active neurons firing at a baseline of 20 Hz. The bottom 66% correspond to silent neurons that fire at 0.017 Hz. Stimulation duration was 200 ms. *C*: optimal learning rate was determined by minimizing root mean square error (RMSE) over 100 iterations for a group of representative recurrent networks and over a range of learning rate values between 10^−5^ and 10^−2^. *D*: derived vs. actual weights in the hyperactive neuron population case (with no silent neurons). The derived weights matched the actual weights (Pearson correlation *r* = 1.00, *P* ∼ 0). *E*: derived vs. actual weights in the realistic neuron population case, where 66% of the population are silent. The derived weights deviated considerably from the actual weights (*r* = 0.44, *P* < 0.01). *F*: derived vs. actual weights in the stimulated population case, where 25% of the population were stimulated to fire at 60 Hz for 100 ms. The derived weights closely matched the actual weights (*r* = 0.97, *P* ∼ 0). *G*: RMSE of the derived weights vs. actual weights for the cases shown in *D–F* (*n* = 10 datasets); error bar is SE.

Δ*w_i_* is the update for input weight *i*, lr is the learning rate, *y* is the spike output of the postsynaptic neuron in the training data, and *y*′ is the output guessed by the perceptron model. We used 2,500 trials (each of 1,000-ms duration) to train the perceptron model with mean optimized learning rate of 0.01 (Fig. 1*F*).

### Performance Measures

We evaluated the performance of the algorithm for deriving connectivity by the root mean square error (RMSE) between the derived weights and actual input weights. In addition, we evaluated performance by using the derived weights to predict spikes, as measured by the average of the prediction sensitivity (true positive rate, TPR) and precision (positive predictive value, PPV):

(*5*) $$\text{TPR}=1-\frac{1}{\left|S_{\text{a}}\right|}\sum_{k\in S_{\text{a}}}s_{k}-{\hat{s}}_{k}\;$$

(*6*) $$\text{PPV}=1-\frac{1}{\left|S_{\text{p}}\right|}\sum_{k\in S_{\text{a}}}s_{k}-{\hat{s}}_{k}$$

where *S*_a_ represents the subset of events in which a postsynaptic spike occurs and *S*_p_ represents the subset of events in which the algorithm predicts there is a postsynaptic spike. *S_k_* is the *k*^th^ event in *S_a_*, and ${\hat{s}}_{k}$ is *k*^th^ event in the corresponding position of predicted spikes. We tested the algorithm performance using 10 randomized simulations, with different spike trains and connectivity. The test data consisted of 2,500 trials of 1000 ms each.

### Stimulated Network Condition

We examined the effect of stimulation on deriving connectivity, setting 50 of the input population spike trains (cells chosen at random) to fire at 60 Hz during each stimulation duration (200 ms) based on commonly used parameters (55). We examined results as a function of the number of stimulations, ranging from 1 to 15.

### Connection Classification

We classified connection types for feedforward networks based on selected ranges (Table 1). We classified weights as unconnected using a small ε (0.16 mV), which was the RMSE of derived weights for actual unconnected neurons in perceptron models that had high accuracy (performance > 0.99). We calculated the accuracy of classification for each connection type as the probability of synaptic weight *w* being correctly classified, given the actual weight.

**Table 1.** Synaptic weight classification

	Strong Inhibitory	Weak Inhibitory	Unconnected	Weak Excitatory	Strong Excitatory
*w* ∈	(−8, −4)	(−4, ε)	(−ε, ε)	(−ε, 4)	(4, 8)

*w*, Synaptic weight; ε, root mean square error (RMSE) of derived weights for actual unconnected neurons in perceptron models that had high accuracy.

### Experimental Data as Model Input

Neural recording data fed as input to feedforward and recurrent networks were acquired from rat cortical neurons (A1084001, lot 2214638, Gibco Thermo Fisher Scientific) cultured on microelectrode arrays (MEAs; 60MEA100/10iR-ITO-gr, Harvard Biosciences) with methods described previously (25, 56). MEAs were coated with 0.1 mg/mL polyethyleneimine (408727, Sigma-Aldrich) and 4 μg/mL laminin (23017-015, Thermo Fisher Scientific). A volume of 50 µL of cells was plated for 4 h at 1 million cells/mL on sterilized MEA in media (Neurobasal Plus + 1× GlutaMAX + 10% fetal bovine serum; Thermo Fisher), maintained in media without serum, and replaced every 2–3 days. Data were recorded between days in vitro (DIV) 18 and DIV 22 with an MZ60 MEA headstage amplifier at 6,104 Hz per channel streamed through PZ5 neurodigitizer amplifier and RZ5P base processor (Tucker-Davis Technologies, Alachua, FL). Total recording duration for each data set was 900 s, beginning with a 60-s stimulation epoch. Stimulation was delivered globally by ambient temperature change (from 37°C to room temperature, 60 s). Raw data files were loaded into MATLAB with TDT Synapse software environment and development kit for processing and analysis.

## RESULTS

### Prediction of Synaptic Weights for Populations with Silent Neurons Is Improved by Stimulation

We characterized the effect of silent neurons on deriving synaptic weights from a heterogeneous population (excitatory, inhibitory, and unconnected) to a postsynaptic neuron within an arena of a recording electrode (Fig. 1, *A* and *B*) and characterized the improvement afforded by stimulation (Fig. 1*C*). Pearson correlation was performed between the inferred weights and the actual weights to quantify the similarity between them. As a reference case, we used a hyperactive neuronal population as a nonrealistic scenario without silent neurons, for which the algorithm derived synaptic weights with high accuracy (Fig. 1*D*; RMSE = 0.01 ± 0.00, Pearson correlation *r* = 1, *P* = 0, *n* = 10 random populations). For the realistic neuron population (with silent neurons), the algorithm failed to predict the weights from silent neurons and also from many of the active neurons (Fig. 1*E*; RMSE = 3.59 ± 0.42, Pearson correlation *r* = 0.44, *P* < 0.01). When the population was sufficiently stimulated, the algorithm was able to derive weights with high accuracy (Fig. 1*G*; RMSE = 0.35 ± 0.06, Pearson correlation *r* = 0.97, *P* ∼ 0, 15 stimulations, 100 iterations; see Supplemental Fig. S1). The stimulation protocol sufficiently improved the weight prediction of a population with silent neurons (Fig. 1*G*) and also for limited fractions of the network recorded as common in experimental configurations (Supplemental Fig. S2; RMSE decreased by 4.12 ± 0.64).

**Figure 2.**
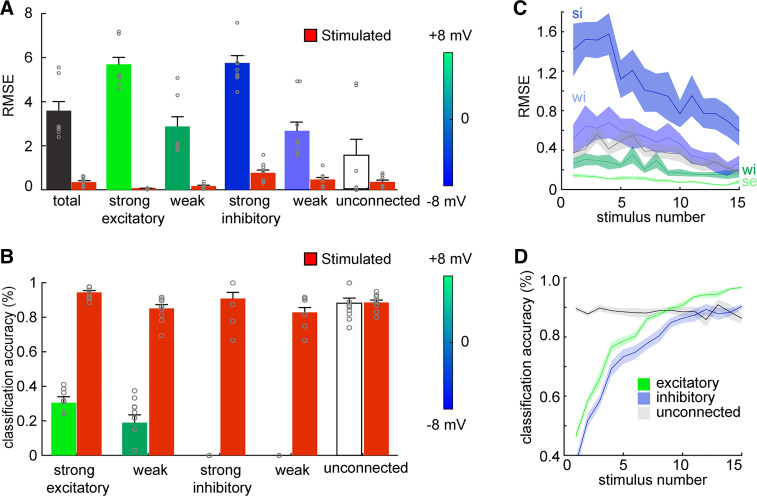
Deriving synaptic weights as a function of stimulus and connection type. *A*: performance of deriving connections of different type (excitatory and inhibitory) and strength (see methods), in the unstimulated and stimulated (red) conditions. Stimulation improved performance for all cases except the unconnected (paired *t* test, *P* < 0.05). RMSE, root mean square error. *B*: classification accuracy for each connection type in the unstimulated and stimulated conditions. *C*: performance of deriving connection weights as a function of stimulus number, for the different connection types as shown in *A*. The shaded area around each curve shows the SD. se, Strong excitatory; si, strong inhibitory; wi, weak inhibitory. *D*: classification accuracy for excitatory, inhibitory, and unconnected neurons as a function of stimulus number. All error bars denote SE (*n* = 10 datasets).

To evaluate the accuracy of inferred weights of different connection types before and after stimulation, we compared the RMSE value of five groups of connections defined according to their type (excitatory or inhibitory) and strength (weak, strong, and unconnected). The RMSE was improved by stimulation for all types of synaptic weights (Fig 2*A*; paired *t* test: *P* < 0.05), except for unconnected cells since their error was small to begin with (*P* = 0.1). Similarly, the classification accuracy of connection types improved by stimulation for all types, especially for inhibitory connections (Fig 2*B*; *P* < 0.05). The classification accuracy of unconnected weights was high in the nonstimulated case and therefore did not improve significantly with stimulation. The accuracy of predicted weights for all connection types improved with increasing the number of stimuli (Supplemental Fig. S1), particularly for strong inhibitory connections (Fig. 2*C*). The classification accuracy for inhibitory and excitatory connections improved drastically with increasing the number of stimuli, because of the activation of silent neurons (Fig. 2*D*).

### Spike Prediction Using the Derived Weights

**Figure 3.**
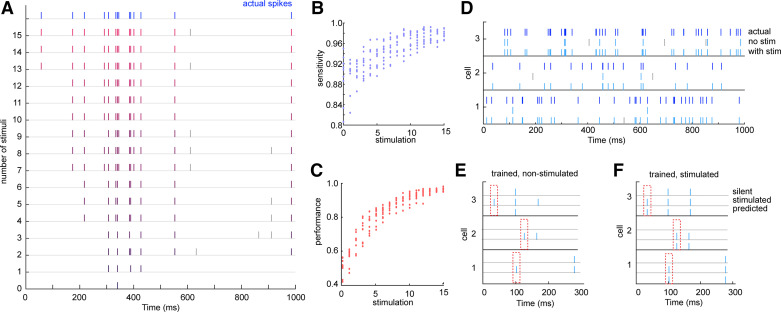
Spike prediction using derived connection weights. *A*: predicted spikes with weights derived using different number of stimuli. The spike train at *top* shows the actual postsynaptic spikes. Listed below are spike trains predicted from populations stimulated with 0–15 stimuli. True positives are shown in blue; false positives are shown in gray. *B*: sensitivity of spike prediction as a function of stimulus number. *C*: precision of spike prediction as a function of stimulus number. *D*: 3 examples of spike trains of the postsynaptic neuron in the test data (*top*) and predicted spikes using derived weights from the nonstimulated population (*middle*) and the stimulated population (*bottom*). Blue spikes are true positives; gray spikes are false negatives. *E*: 3 examples of spike trains of the postsynaptic neuron in the nonstimulated case (*top*) and when stimulating 1 strong excitatory silent neuron (*middle*) and the predicted spikes using weights derived from the nonstimulated population data (*bottom*). *F*: same as *E* but with predicted spikes using weights derived from the stimulated population data.

We validated the performance of the derived connection weights from unstimulated and stimulated populations in predicting spikes of the postsynaptic neuron, using test data of the population spikes in the stimulated condition (Fig. 3*A*). Weights derived from nonstimulated populations predicted spikes poorly, with a high proportion of false negatives and some false positives. In contrast, derived weights from stimulated populations predicted postsynaptic spikes with high accuracy, with the sensitivity and performance of the model reaching 0.96 ± 0.01 (Fig. 3*B*) and 0.97 ± 0.01 (Fig. 3*C*) after 15 stimuli, respectively. We also verified that the derived weights performed well on population data from the fully activated condition (Supplemental Fig. S3). To verify that the stimulation of silent neurons significantly affects the postsynaptic spike pattern, we stimulated a single strong excitatory silent neuron and compared the predicted postsynaptic spikes, using weights derived from nonstimulated and stimulated data. Activating the single silent neuron was sufficient to trigger additional spikes (Fig. 3*E*, dashed box), which were not predicted when using weights derived from nonstimulated population data (Fig. 3*E*, silent) but were predicted faithfully using weights derived from stimulated population data (Fig. 3*F*, stimulated).

### Stimulated Recurrent Circuits

**Figure 4.**
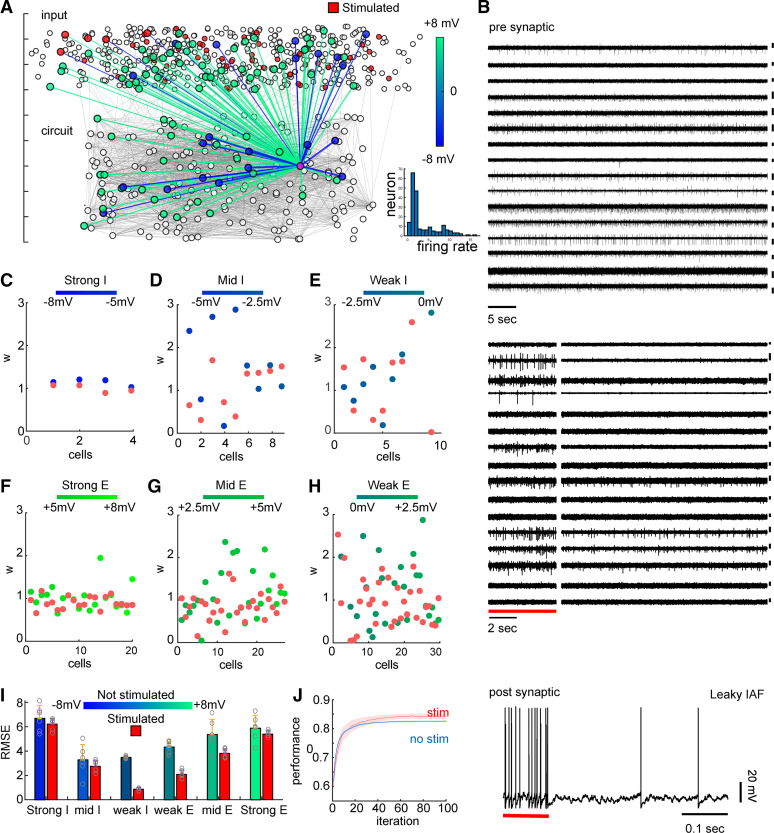
Application of learning algorithm to realistic circuit with live neuronal recordings. Neural network reconstruction is improved with supervised learning of a stimulated network of leaky integrate-and-fire (IAF) neurons fed with live neural recordings. *A*: circuit configured with 4-to-1 excitatory-to-inhibitory ratio and probabilities *P* = 0.13 for unidirectional connections and *P*_rep_ = 0.06 for reciprocal connections. *B*: recordings fed to network. Shown are 16 channels, 60 s each, of a total of 64 channels, 900 s each fed to circuit as input (scale bar is 100 µV). *C–H*: improved prediction of connectivity for strong/mid/weak inhibitory (I) and excitatory (E) subpopulations divided by agglomerative clustering (Δ*w* = difference between derived and real weights) *I*: root mean square error (RMSE) of weight prediction for different subpopulations. *J*: algorithm performance with and without stimulation (red and blue, respectively) for 100 iterations. Algorithm was trained on all data indiscriminately. All error bars denote SE (*n* = 6 datasets).

To evaluate performance in a realistic scenario, we tested the algorithm on recurrent circuits of leaky integrate-and-fire (LIF) neurons, receiving real experimental data as presynaptic input (Fig. 4). The circuits were constructed as interconnected hubs with both unidirectional and reciprocal connections and typical synaptic strength (52, 57) (Fig. 4*A*) and displayed lognormal firing rate distributions (Fig. 4*A*, *inset*). A postsynaptic cell received input from six datasets of 64-channel single-unit extracellular microelectrode array recordings, each with a total duration of 900 s (Fig. 4*B*). Then the model was trained to estimate the synaptic connections between neurons. Decrease in the difference between derived and real weights (Δ*w*) was observed to variable degrees within six subpopulations (strong, medium, and weak inhibitory/excitatory; see for example Fig. 4, *C–E* and *F–H*, respectively). This effect was observed across all datasets (Fig. 4*I*), with significant improvement seen in weak inhibitory, weak excitatory, and medium excitatory (*P* < 0.015, *n* = 6). Performance for a feedforward LIF network receiving experimental data as input was also improved when the algorithm was trained on 60-s stimulated multichannel data and tested on 840 s (Fig. 4*B*).

### Large Neuronal Populations

**Figure 5.**
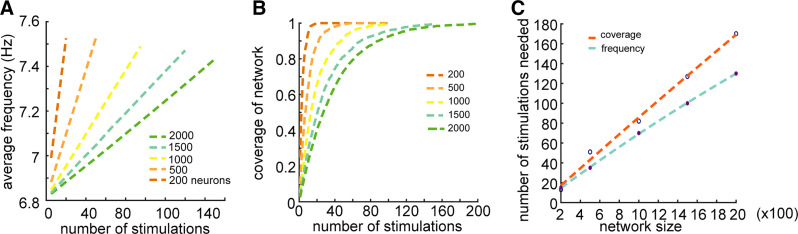
Deriving connectivity for large-scale networks. *A*: average spike frequency as a function of the number of stimulations for different network sizes. The frequency increased linearly with the number of stimulations. The slope decreased as the network size increased. *B*: coverage of the population by stimulation, measured by % of the population activated, as a function of stimulus number. The slope of the curve decreased as the size of network increased. *C*: the number of stimulations needed to reach 99% coverage increased linearly with network size. Similarly, the number of stimulations needed to reach a frequency of 7.4 Hz (corresponding to the 200 neurons, 15 stimuli case) increased linearly with network size.

To determine the data requirements for deriving connection weights from larger neuronal populations, we calculated the average spike rate as an estimate of the number of spikes in the data from populations of sizes ranging from 200 to 2,000 neurons (Fig. 5). The average spike rate increased linearly with the number of stimulations, and the slope of that relationship decreased with network size (Fig. 5*A*). The number of stimuli necessary to yield a firing rate similar to that in the 200-neuron population with 15 stimuli increased linearly with population size (Fig. 5*C*). We next estimated the degree coverage afforded by the stimuli in populations of different size, as measured by the percentage of neurons that get stimulated by the random stimuli overall (Fig. 5*B*). The number of stimuli necessary to get 99% coverage increased linearly with network size (Fig. 5*C*), so that <200 stimulations were sufficient to get full coverage for all the populations examined.

## DISCUSSION

In this study, we demonstrated the utility of stimulation for deriving connectivity of neuronal populations with a significant proportion of the network comprising silent neurons, as seen physiologically. To date, the challenge imposed by silent neurons on deriving connections has remained largely unexplored. Here we showed that a supervised learning algorithm combined with a small number of stimulations enabled inferring connection weights with high fidelity and predicting spike trains with high accuracy. We have characterized the challenge pressed by silent neurons for different types of connections and for different population sizes. Our results make testable predictions about the number and protocol of the required stimulations, which is expected to enhance future efforts for deriving neuronal connectivity that underlies brain function.

We applied our method on heterogeneously interconnected LIF circuits receiving live multichannel neural recordings as input and demonstrated improved performance during stimulation for multiple subpopulations. Implementation of the method for diverse electrophysiological datasets relies on using experimental parameters for selective and continuous stimulation of interconnected neuronal subpopulations. Multiplexed single-spike acquisition of connected networks during stimuli is routine both clinically and preclinically over multiple hours during different forms of stimuli (15, 22, 25). Robust changes in firing rates are prevalent during both sensory and electrical stimuli, but fine-tuning of firing patterns with only modest changes in firing rates is also possible. Recent work demonstrates modulation of subtle features of cortical activity including phase locking and interspike intervals of subpopulations, with only minor changes in firing rates (58). Future expansion of our method will involve burst response during stimulation that emphasizes changes in spiking patterns over increase in rate, to identify scenarios that sufficiently add to the quantity of information needed for deriving weights and predicting spikes. Moreover, our simulations suggest the ability to predict subtle changes in firing rates of up to single spikes evoked by single presynaptic cells (Fig. 3, *E* and *F*). In our circuit-level validation using live neuronal MEA recordings we show that prediction of synaptic weights can be improved by stimulation (Fig. 4). Further work using more realistic in vivo datasets and circuits combining both feedforward and feedback circuits can address this challenge of testing whether single spikes can also be predicted in scenarios where the number of neurons for a given network is largely unknown. This offers a way to reconstruct networks containing neurons with highly selective receptive field that can also respond to abstract stimuli, a cell type seen physiologically in animals and humans in the form of place cells and abstract concept cells (59).

Our tests involve bringing a portion of the population to above firing baseline as a basis for deriving connectivity. Some stimulation experiments, particularly involving deep brain electrical stimulation for neurotherapeutic purposes, result in reduction of firing rates toward complete inhibition in some regions and sometimes generate a combination of excitation and inhibition in response to stimulus (8, 60). The paradigm we present here assumes targeting of a brain region excitatory to the network studied. This can in turn drive the design of new experiments for characterizing mesoscale connectivity of anatomical regions in the brain while also facilitating maximization of the amount of information acquired about the network. Recent large-scale recordings in awake rodents provide a viable platform for stimulus-derived connectivity mapping, demonstrating the activation of subpopulations across multiple brain regions, spanning the primary visual cortex, hippocampal, and thalamic regions in response to constant visual stimulus (29). Such increasingly accessible large-scale tools for electrophysiology will permit methodically locating excitable regions at single-cell and single-spike resolution across the brain. Consequently, our results affirm that local electrical stimulation is not likely to facilitate network reconstruction, in large part because of indiscriminate synchronous firing and false positive observations that lead to imprecise determination of synaptic weights (Supplemental Fig. S4). This coincides with known constraints related to the symmetry of the radius of influence surrounding an electrode (61) that result in indiscriminate stimulation at the recorded region and will likely obscure connectivity manifested in spiking patterns. For preclinical research, however, where optogenetic stimulation is available at the single-cell type level (62), our results should lend themselves well where there is a high degree of control of the number and volumetric distribution of stimulated neurons. For reconstruction of large-scale networks, we find that longer stimulation sessions are required, with a linear relationship between network size and number of stimulation epochs for sufficiently precise derivation of synaptic weights (Fig. 5). Long-term changes in network connectivity during training over many days (63) suggest that future studies involving very large-scale electrophysiological recording and long stimulation sessions, network habituation, and rewiring will have to be included in the derivation algorithm.

We characterized the use of stimulation to derive connections from a population of neurons onto a postsynaptic neuron. This enabled a focused investigation of the issue of silent neurons in deriving connections. Our results and stimulation framework can be used to expand the investigation into deriving connections from recurrent networks and overcome additional issues such as spurious connections due to spike correlations (64). We employed an integrate-and-fire neuron model with simple implementation of synaptic connections. Although such models are used widely to model brain networks (65), it will be of general interest to apply our stimulation framework to derive connections from ground-truth spiking data obtained from networks with detailed biophysical models of neurons and synaptic dynamics (50, 57, 66). We anticipate that overcoming the nonlinear challenges imposed by realistic neuronal networks will involve using our framework in tandem with methods for deriving connections based on spike cross-correlations (15, 22) or by using general-purpose optimization methods such as genetic algorithms or maximum likelihood (16, 67).

### Conclusions

We established stimulation parameters for deriving connectivity of silent neuronal populations by way of a supervised learning algorithm. Our algorithm provides network reconstruction with high fidelity with accurate spike prediction. We characterized performance for different connection types and population sizes and verified improved predictions for realistic circuits receiving live neural recording as input. This work establishes a method that is expected to enhance future efforts for deriving neuronal connectivity underlying brain function (68).

## DATA AVAILABILITY

Data will be made available upon reasonable request.

## SUPPLEMENTAL MATERIAL

10.5281/zenodo.7929987 Supplemental Figs. S1–S4: https://doi.org/10.5281/zenodo.7929987.

## GRANTS

This research was funded by NIH Grants K01EB027184 and DP2NS122605 to A.H. This material is also based upon research supported by the US Office of Naval Research under PANTHER award numbers N00014-23-1-2006 and N00014-22-1-2371 to A.H. through Dr. Timothy Bentley.

## DISCLOSURES

No conflicts of interest, financial or otherwise, are declared by the authors.

## AUTHOR CONTRIBUTIONS

X.R. and A.H. conceived and designed research; X.R. and I.B. performed experiments; X.R., I.B., and A.V. analyzed data; X.R., A.V., and A.H. interpreted results of experiments; X.R. prepared figures; X.R. drafted manuscript; X.R. and A.H. edited and revised manuscript; A.H. approved final version of manuscript.
